# Inhaled Antibiotics for the Prevention of Respiratory Tract Infections in Children With a Tracheostomy

**DOI:** 10.3389/fped.2021.633039

**Published:** 2021-02-05

**Authors:** Camille Jutras, Julie Autmizguine, Maryline Chomton, Christopher Marquis, The Thanh-Diem Nguyen, Nadia Roumeliotis, Guillaume Emeriaud

**Affiliations:** ^1^Department of Pediatrics, Centre Hospitalier Universitaire Sainte Justine, Université de Montréal, Montréal, QC, Canada; ^2^Department of Pharmacology and Physiology, Université de Montréal, Montréal, QC, Canada; ^3^Research Center, Centre Hospitalier Universitaire Sainte-Justine, Université de Montréal, Montréal, QC, Canada; ^4^Department of Pharmacy, Centre Hospitalier Universitaire Sainte Justine, Université de Montréal, Montréal, QC, Canada

**Keywords:** ventilator-associated pneumonia, infection, inhaled antibiotics, tracheostomy, pediatric

## Abstract

**Objective:** To describe the use of prophylactic inhaled antibiotics in children with a tracheostomy and assess if its use is associated with a reduction in exposition to broad-spectrum antibiotics and a lower risk of acquired respiratory tract infections.

**Methods:** A case series study was performed in a tertiary care university affiliated hospital. All consecutive children (<18 years old) with a tracheostomy, hospitalized between January 2004 and November 2016, and treated with prophylactic inhaled antibiotics were identified. We analyzed the 3 month- period before and after initiation of prophylactic inhaled antibiotics and described exposure to broad spectrum antibiotics, the number of respiratory tract infections and the associated adverse events.

**Results:** Six children (median age: 11 months, range: 8–100) were included. One received colimycin, 3 received tobramycin and 2 were treated with both antibiotics in alternance. The median duration of treatment was 74 days (22–173) with one patient still being treated at the end of the study. Patients were exposed to systemic antibiotics for 18 days (2–49) in the 3 months preceding the treatment vs. 2 days (0–15) in the 3 months following the treatment initiation (*p* = 0.115). The number of respiratory tract infections went from median of 2 (0–3) to 1 (0–1) during the same periods (*p* = 0.07). Adverse events most commonly reported were cough (*n* = 2) and increased respiratory secretions post-inhalation (*n* = 4). Only one new bacterial resistance was observed.

**Conclusions:** This series of consecutive cases underlines the need for future studies evaluating the potential benefit of prophylactic inhaled antibiotics in children with a tracheostomy.

## Introduction

Tracheostomized children are at increased risk of bacterial tracheostomy-associated respiratory tract infections (bTARTIs) such as Ventilator Associated Pneumonia (VAP), pneumonia, tracheitis and bronchitis (1, 2). The tracheostomy tube bypasses the natural protective barrier of the respiratory tract allowing bacteria to enter the respiratory tract. Recurrent respiratory tract infections in these children result in frequent hospitalizations, longer hospital length of stay, and increased exposure to broad-spectrum antibiotics leading to a greater risk for antibiotic resistance, and important costs to the health care system (2–5).

Preventive measures demonstrated to reduce the occurrence of VAP in the Pediatric Intensive Care Unit (PICU) include bundles of interventions such as hand hygiene, elevation of the head of bed, daily sedation interruption, and daily assessment of extubation readiness (6–8). Despite these, VAP is still frequent, and focus is shifting to other measures such as the use of preventive inhaled antibiotics. Preventive inhaled antibiotics were first used in patients with cystic fibrosis and bronchiectasis, in order to reduce both the bacterial load in secretions and the rate of respiratory exacerbations. Their use in cystic fibrosis patients, most commonly with tobramycin and colimycin, is associated with improved pulmonary function (9–12). In the adult ICU population, preventive inhaled antibiotics have been shown to be effective in decreasing the rate of VAP and decreasing airway colonization, without evidence of more infections with multi-resistant pathogens (13–19).

The preventive use of inhaled antibiotics in the pediatric tracheostomized population to decrease VAP or other type of bTARTIs has not been reported. Given the benefits of inhaled antibiotics in adults and in cystic fibrosis patients, and their safety profile, preventive inhaled antibiotics have been used in our hospital to prevent recurrent respiratory tract infections in patients with tracheostomy.

The goal of this study is to describe the use of inhaled prophylactic antibiotics in hospitalized children with tracheostomy in our institution, and to evaluate whether this intervention reduces exposure to systemic broad-spectrum antibiotics and the occurrence of respiratory tract infections. Our hypothesis is that a preventive course of inhaled antibiotics is associated with a reduction in systemic broad-spectrum antibiotic exposure and in community and hospital-acquired respiratory tract infections.

## Methods

We performed a retrospective case series of consecutive patients. Eligible children were aged 0–18 years old, with a tracheostomy, hospitalized at the CHU Sainte Justine between January 2004 and November 2016. From these patients, we identified those treated with preventive inhaled antibiotics (tobramycin or colimycin), and reviewed charts to confirm inclusion. We excluded patients with cystic fibrosis, children for whom inhaled antibiotics were prescribed for the treatment of an infection, as well as children followed in other centers after being discharge from our institution (missing outcome data).

For each included patient, we collected demographic information. The outcomes were evaluated in the 3-month period before and after the beginning of the inhaled antibiotics with the exception of development of bacterial resistance which was evaluated in the 12-month period after the beginning of this preventive therapy. If patients were discharged home before the end of the study period, we evaluated outcomes based on the endotracheal cultures and completion of the chart that were performed in the outpatient hospital clinics during routine visits.

Our primary outcome was exposure to systemic broad-spectrum antibiotics (IV or PO) in the 3-month period before and after inhaled prophylactic antibiotics. A 3-month period pre- and post-intervention was chosen to allow sufficient time to evaluate the treatment effect, while being short enough to minimize confounding related to changes in the patient's conditions and age. We defined systemic broad-spectrum antibiotics as ampicillin-clavulanic acid, piperacillin-tazobactam, ticarcillin, ticarcillin-clavulanic acid, 3rd and 4th generation cephalosporins, carbapenems, fluoroquinolones, and vancomycin, which could have been given for reasons other than respiratory tract infections. This outcome was chosen as an objective measure given the risk of missing undocumented respiratory tract infections. We also evaluated antibiotic exposure in terms of antibiotic-days, measured as the number of antibiotics received on a given day multiplied by number of days of treatment. This outcome was used as exposure to more than one systemic antibiotic increases cost, risk for side effects and bacterial resistance.

As secondary outcomes, we collected the number of respiratory tract infections, defined as pneumonia, tracheobronchitis or VAP diagnosed by the attending physician, as well as the acquisition of newly resistant bacterial strains after the initiation of inhaled antibiotics. The emergence of newly resistant bacterial strains was defined as documented bacteria in the endotracheal secretions, in the 12 months following prophylactic inhaled antibiotics, which was resistant to the previously prescribed inhaled antibiotics or to any other broad-spectrum antibiotics as defined above. This included a change in the sensitivity profile for tracheal bacteria before the inhaled antibiotics (newly resistant), or the appearance of newly resistant bacterial strains. Evaluation of development of resistance over a longer period than other outcomes was chosen not to miss any new resistance that could have appear after 3 months as treatment with inhaled antibiotics can be given for a long period of time, and because endotracheal cultures were frequently obtained after the 3-month period. The characteristics of the inhaled antibiotic prophylactic course were also collected, as well as adverse events reported in the chart.

Categorical variables were reported with absolute numbers and percentages. Continuous variables were reported using medians with range (min-max). Between-period differences were tested using a Wilcoxon rank test (continuous variables) or a Fisher exact test (categorical variables). A *p*-value < 0.05 was considered significant. Statistical analysis was performed using SPSS Statistics 23 (IBM). The study was approved by the local Research Ethics Committee with a waived consent.

## Results

We identified 17 eligible children through cross verification of medical archives and the pharmacy database. After the chart review, 6 of them (4 males) were included in this case series. Eleven children were excluded as inhaled antibiotics were used as treatment for active infection (*N* = 5), or the follow-up after hospitalization was in another center (*N* = 6). Table 1 details baseline characteristics and treatment description for children included. Median age at the beginning of the prophylactic treatment was 11 months (range 8–100). Three children (50%) had a tracheostomy for upper airway disease (severe tracheobronchomalacia, subglottic stenosis, massive hypertrophic arythenoid) and the three others for ventilator dependency (secondary to bronchopulmonary dysplasia; diaphragmatic paralysis and intensive care myopathy; multifactorial chronic respiratory insufficiency, respectively). Inhaled antibiotics were initiated for the majority of children (4/6) during the same hospitalization as the tracheostomy. Four children received their full course of preventive inhaled antibiotics while in the hospital, and two children were discharged home with inhaled antibiotics. The median duration of inhaled antibiotics was 74 days (range 69–173) with one child still on this prophylaxis at the end of the study. Three children received inhaled tobramycin (80 mg BID), 1 received inhaled colimycin (75 mg BID) and 2 children received both antibiotics in alternance.

**Table 1:** Baseline characteristics of the cases and description of the inhaled antibiotic treatments (*n* = 6).

Age at the beginning of the treatment (months)	11.0 (8.0–100.0)
Weight (kg)	9.1 (6.3–24.0)
Sex (male)	4 (67%)
**Reason for the tracheostomy**	
Upper airway causes	3 (50%)
Ventilator dependence	2 (33%)
Bronchopulmonary dysplasia	1 (17%)
**Number of days between the tracheostomy and the beginning of the antibiotic treatment**	101 (48–2731)
**Preventive inhaled antibiotic treatment length (in days)**^**a**^	74 (22–173)
**Type of inhaled antibiotic**	
Tobramycin only (80 mg BID)	3 (50%)
Colimycin only (75 mg BID)	1 (17%)
Both (alternately)	2 (33%)
**Hospital location at the beginning of the treatment**	
Pediatric intensive care unit (PICU)	5 (83%)
Pediatric ward	1 (17%)
**Type of ventilation at the beginning of treatment**	
Continuous mechanical ventilation	3 (50%)
No ventilation	2 (33%)
Nocturnal mechanical ventilation	1 (17%)
**Adjuvant treatment**	
Salbutamol	5 (83%)
None	1 (17%)
**Adverse events**	
Increased respiratory secretions	4(67%)
Cough	2 (33%)
Respiratory deterioration at the time of treatment	1 (17%)
Bronchospasm	0 (0%)
**Length of stay (days)**	
PICU stay^b^	283 (4–426)
Hospital stay	345.5 (6–771)

*Variables are reported as median (range) or number (percentage)*.

a *One patient was excluded from this median since treatment is still ongoing at the end of the study period*.

b *One patient was never hospitalized in the pediatric intensive care unit*.

**Figure 1:**
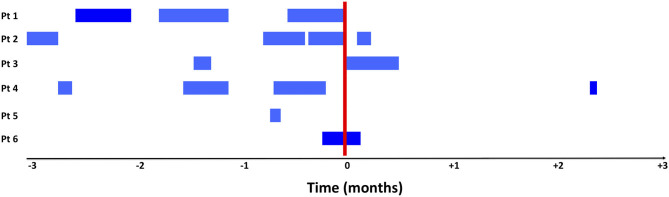
Exposure to systemic broad spectrum antibiotics in the 3-month period before and after initiation of treatment with inhaled antibiotics. The blue bars illustrate all days with an exposure to broad spectrum systemic antibiotics and the red bars represent the start of inhaled antibiotics. Inhaled antibiotics were started for patient 1 and 2 after a systemic course of antibiotics for a respiratory infection. Patient 6 was on systemic antibiotic for an active respiratory infection when preventive inhaled antibiotics were started.

The outcomes are presented in Table 2. The children were exposed to systemic broad-spectrum antibiotics for a median of 18 days (range 2–49) in the 3 months preceding prophylactic inhaled antibiotics, vs. 2 days (0–15) in the 3 months following the prophylactic inhaled antibiotics (*p* = 0.115). The number of respiratory tract infections went from a median of 2 (0–3) to 1 (0–1) in the same periods, respectively (*p* = 0.07). Figure 1 illustrates broad-spectrum antibiotic treatment before and after the beginning of prophylactic inhaled antibiotics. Inhaled antibiotics were initiated in two children at the end of a course of systemic antibiotics and in one child while he was on systemic antibiotics for a respiratory infection. All six patients had received systemic antibiotics in the index hospitalization.

**Table 2:** Infectious outcome measures in patients before and after inhaled antibiotics.

	**3 months before initiation of inhaled ATB**	**3 months after initiation of inhaled ATB**	***p* value**
Days of systemic (PO or IV) antibiotic exposure	18 (2–49)	2 (0–15)	0.115
Antibiotic exposure (antibiotic-days)^a^	23 (2–85)	2 (0–15)	0.116
Number of antibiotic-treated respiratory tract infections	2 (0–3)	1 (0–1)	0.066
**Stated reason for discontinuation of prophylaxis**			
Discontinuation of ventilation		2 (33%)	
Decanulation of the tracheostomy		1 (17%)	
End of the planned prophylaxis	N/A	1 (17%)	N/A
Not mentioned		1 (17%)	
Still ongoing		1 (17%)	
**Type of bacteria detected in endotracheal secretions**	6 (100%)	4/5 (80%)^b^	N/A
*Pseudomonas aeruginosa*	4	2	
*Staphylococcus aureus*	1	2	
*Enterobacter cloacae*	2	0	
*Klebsiella pneumoniae*	3	0	
*Serratia marcescens*	2	0	
*Haemophilus influenzae*	2	1	
*Stenotrophomonas maltophilia*	1	1	
*Enterococcus fecalis*	0	1	
*Proteus Mirabilis*	0	1	
Development of bacterial resistance	N/A	1/5^c^	N/A

*ATB, Antibiotic; po, oral; IV, intravenous; N/A, Not applicable*.

*Variables are reported as median (range) or number (percentage)*.

a *Measured as the number of antibiotics received by the patient on a specific day multiplied by number of days of treatment*.

b *Evaluated in the 12 months period after the beginning of the treatment with inhaled antibiotics. One patient had no endotracheal culture after the beginning of the treatment*.

c *Positive endotracheal culture with Stenotrophomonas maltophilia resistant to ceftazidime*.

Adverse events of the inhaled antibiotics commonly reported were increased respiratory secretions (*N* = 4) and cough (*N* = 2). No bronchospasm was reported. In one case, a respiratory deterioration occurred after the treatment: the patient coughed, then desatured, requiring increased oxygen for an 8-h period.

In the 3-month period before initiation of the inhaled antibiotics, all six (100%) children had a positive endotracheal culture for at least one bacteria. In the 12 months following treatment initiation, endotracheal bacteria were documented in only four children. A newly resistant bacterial strain was observed in one child (Table 2) while receiving inhaled tobramycin; a positive endotracheal culture with *Stenotrophomonas maltophilia* resistant to ceftazidime, a resistance which is frequent and unlikely linked to inhaled antibiotics.

## Discussion

This case series of hospitalized children with tracheostomy describes the use of prophylactic inhaled antibiotics, the subsequent reduction in exposure to systemic broad-spectrum antibiotics and respiratory tract infections, with infrequent emergence of newly resistant bacterial strains despite a prolonged inhaled antibiotic use. The use of inhaled antibiotics in this population remains low locally, as we were unable to achieve our target sample size to power the study and established the significance of the findings.

Children with tracheostomy usually have lengthy hospitalization and often require prolonged mechanical ventilation. A tracheostomy is an independent risk-factor for VAP (6, 7) contributing to morbidity and mortality, weaning difficulty, increased exposure to large-spectrum antibiotics, promoting microbial resistance and increased healthcare costs (3, 4). Regardless of ventilation, a tracheostomy increases the risk of respiratory tract infections resulting in frequent hospitalizations and increased exposure to systemic broad-spectrum antibiotics (1, 2, 5).

Prophylactic inhaled antibiotics offer some theoretical benefits. First, local drug delivery is maximized, and systemic side effects such as *C.difficile* infection, are limited. Second, antibiotic coverage with tobramycin and colimycin is adequate for the majority of bacteria causing VAP and hospital acquired infections. These antibiotics are not typically first line therapy in the standard systemic treatment of VAP, which reduces the risk of developing antibiotic resistance against them. Third, prophylactic inhaled antibiotics have been shown to reduce morbidity in adult ICU patients (14–16). Last, inhaled antibiotics reduce lung inflammation in patients with lung disease (bronchopulmonary dysplasia or ventilation induced lung injury). In critically ill ventilated patients, lung bacterial burden and presence of gut-associated bacteria is associated with poorer ICU outcomes (20). As described in bronchiectasis and cystic fibrosis patients, inhaled antibiotics could contribute to reduce bacterial burden, chronic colonization, and inflammation therefore improving lung function (9, 21). On the other hand, it could also disrupt the lung microbiota and contribute to the lung disease. Evaluation of the effect of preventive inhaled antibiotics on the lung microbiota of tracheostomy's patient needs to be further investigated, as well as the impact of the bacterial load on the response to this therapy.

In our study, patients received inhaled colimycin and/or tobramycin, were treated for a median of 74 days, and the treatment was stopped most frequently when the ventilation was discontinued. These characteristics are similar to what has been described in the adult population. Tobramycin (or gentamicin) and colimycin are frequently used in adult studies for the prevention of VAP, with literature reporting various dosing and duration of treatment. Depending on the studies, inhaled antibiotics are given twice or three times daily, for a duration varying between 7 days and time to extubation (15). The optimal length of therapy and the criteria to initiate and cease preventive inhaled antibiotics are still unknown.

We chose to evaluate use of systemic broad-spectrum antibiotics because it is a clinically relevant, easy to measure, objective outcome. This outcome however, is not solely associated with the exposure to inhaled antibiotics and can reduce exposure effects as broad-spectrum antibiotics may have been given for reasons other than a respiratory infection. We also cannot rule out a potential detection bias, linked to the fact that systemic antibiotic initiation may have been delayed or postponed, because of the presence of inhaled antibiotics. Despite this, our results are consistent with the evolution of the number of respiratory tract infections. Diagnostic bias is also possible given the fact that diagnosis of respiratory tract infections was collected based on subjective criteria (physician judgment) rather than objective criteria. In addition, improvement of the underlying disease or patient growth could have resulted in a lower rate of acquired respiratory tract infections or reduced treatment rate, although the temporal trend observed in Figure 1 seems more in favor of a response to the intervention.

There are limitations to this study. The sample size was smaller than expected. Due to the retrospective design, the assessment of outcomes was dependent on medical chart interpretation. Therefore, we cannot exclude that some data, in particular the identification of adverse events, may not be exhaustive. All children received systemic antibiotics for a respiratory infection in the index hospitalization; this could be confounding as this exposure was included in the assessment of antibiotic exposure in the pre- and post-periods. Furthermore, the decision to start inhaled preventive antibiotics was not standardized or always well documented, introducing a possible selection bias. Included patients varied in terms of clinical characteristics (ventilation) resulting in a heterogenous population. We also had to exclude several children because the initiation of inhaled antibiotics was not sufficiently documented and prevented the determination of the outcomes of interest.

## Conclusion

This case series is the first to describe the preventive use of inhaled colimycin or tobramycin in children with a tracheostomy. We observed a non-significant decrease in exposition to broad-spectrum antibiotics and the incidence of acquired respiratory tract infection, without any significant adverse events or emergence of significant bacterial resistance. This study supports the need for further studies evaluating the potential benefits of prophylactic inhaled antibiotics in tracheostomy patients.

## Data Availability Statement

The raw data supporting the conclusions of this article will be made available by authors upon reasonable request and after approval by the ethics committee of CHU Sainte Justine.

## Ethics Statement

The studies involving human participants were reviewed and approved by the ethics committee at CHU Sainte-Justine. Written informed consent from the participants' legal guardian/next of kin was not required to participate in this study in accordance with the national legislation and the institutional requirements.

## Author Contributions

CJ and GE designed the study and wrote the protocol which was approved by JA, MC, CM, TN, and NR. CJ performed data collection, data analysis, and wrote the manuscript in collaboration with GE. JA, MC, CM, TN, and NR reviewed and approved the final proof of the manuscript. All authors contributed to the article and approved the submitted version.

## Conflict of Interest

The authors declare that the research was conducted in the absence of any commercial or financial relationships that could be construed as a potential conflict of interest.
